# Altered interictal alpha-band organization in childhood absence epilepsy

**DOI:** 10.3389/fninf.2026.1875377

**Published:** 2026-10-23

**Authors:** Miroslaw Latka, Pawel Glaba, Marta Kuryło, Beata Jędrzejczyk-Góral, Wojciech Walas, Sławomir Kroczka, Justyna Paprocka

**Affiliations:** 1 Department of Biomedical Engineering, Wroclaw University of Science and Technology, Wrocław, Poland; 2 Department of Pediatric Neurology, T. Marciniak Hospital, Wrocław, Poland; 3 Department of Child Neurology, "Promyk Słońca" Diagnostic and Rehabilitation Center, Wroclaw, Poland; 4 Neonatal and Pediatric Intensive Care Unit, University Hospital in Opole, Opole, Poland; 5 Department of Child Neurology, Jagiellonian University Medical College, Kraków, Poland; 6 Department of Neurology, Upper Silesian Child Health Center, Silesian Medical University, Katowice, Poland

**Keywords:** alpha rhythm, childhood absence epilepsy (CAE), phase-lagged synchronization, surrogate analysis, weighted phase-lag index

## Abstract

**Objective:**

Interictal background EEG in childhood absence epilepsy (CAE) may appear normal. We asked whether drug-naive children with CAE differ from controls in alpha frequency and the regional distribution of phase-lagged synchronization, and how these differences depend on participant-specific surrogate baselines and analysis frequency.

**Methods:**

We analyzed eyes-closed resting EEG (19 channels, 26 s) from 49 drug-naive children with CAE and 83 controls. Individual alpha frequency (IAF) was estimated by spectral parameterization of posterior channels. Weighted phase-lag index (wPLI) at 12 Hz was summarized globally and as the frontal–posterior log-ratio λ_FP_ = ln(wPLI_F_/wPLI_P_), where F and P denote frontal and posterior within-region means. Participant-specific baselines were obtained from 100 circular-shift surrogates, preserving each channel's wavelet-coefficient sequence up to circular translation while altering inter-channel alignment. Group comparisons used studentized permutation tests. The regional contrast was also evaluated at each participant's IAF.

**Results:**

IAF was lower in patients (8.80 versus 9.42 Hz; *p* = 0.002). At 12 Hz, controls showed posterior predominance of synchronization (λ_FP_ = −0.352), which was largely removed by circular shifting (−0.036), whereas patients' mean regional balance was near zero. After baseline subtraction, the patient-minus-control difference in λ_FP_ was 0.332 (95 % CI [0.181, 0.483]; *p* < 0.001; Hedges' *g* = 0.79). In the 131 participants with identifiable IAF, the contrast was 0.316 at 12 Hz and 0.124 at IAF (*p* = 0.139). A direct comparison using within-participant changes supported a larger contrast at 12 Hz (difference 0.192, 95 % CI [0.017, 0.367]; exploratory, unadjusted *p* = 0.032). The global wPLI difference after baseline subtraction remained uncertain (−0.021, 95 % CI [−0.044, 0.001]; *p* = 0.069).

**Conclusions:**

Drug-naive CAE was associated with slower alpha activity and reduced posterior predominance of phase-lagged synchronization at 12 Hz. Circular shifting largely removed the controls' posterior predominance, supporting a difference in regional organization sensitive to inter-channel alignment. The smaller contrast at IAF indicates that analysis frequency materially affects this finding, without establishing that alpha slowing alone explains it.

## 1 Introduction

Conventional EEG assessment in epilepsy focuses primarily on interictal epileptiform discharges, which are diagnostically valuable but provide limited information about persistent alterations in the background activity between seizures (Koutroumanidis et al., 2017). Quantitative EEG can complement this assessment by characterizing oscillatory frequency, spectral composition and inter-regional relationships, and has therefore been widely investigated as a source of interictal biomarkers of disease state, treatment response and outcome (Engel Jr et al., 2013; Gallotto and Seeck, 2023).

Childhood absence epilepsy (CAE) is a useful model in which to study such alterations. Typical absence seizures are accompanied by generalized rhythmic spike-and-wave discharges and marked transient synchronization of distributed thalamocortical networks (Crunelli et al., 2020; Hirsch et al., 2022). Outside seizures, however, routine EEG may appear visually unremarkable. This is clinically relevant because CAE is not uniformly benign: treatment failure is common, and attentional and other neurobehavioral difficulties may persist despite seizure control (Glauser et al., 2013; Masur et al., 2013). Persistent interictal network abnormalities may therefore capture information that is not represented by seizure counts alone.

Network involvement in absence epilepsy is spatially non-uniform. Experimental models support focal cortical initiation of spike-and-wave activity followed by rapid recruitment of thalamocortical circuits (Meeren et al., 2002; Polack et al., 2007). Human EEG-fMRI and MEG studies likewise indicate regional differentiation during seizures, including frontal and parietal involvement and anterior–posterior reorganization of alpha activity (Bai et al., 2010; Li et al., 2025). In the interictal state, a disturbance of directed anterior–posterior connectivity has been reported in drug-naive children with CAE (Rotondi et al., 2016). These observations motivate an analysis that considers not only global synchronization, but also how synchronization is distributed between anterior and posterior scalp regions.

The interictal alpha rhythm may additionally differ in frequency. Alpha activity becomes faster during childhood and adolescence (Srinivasan, 1999; Uhlhaas et al., 2010), whereas slowing of alpha activity has been reported in epilepsy (Abela et al., 2019). Medication is a potential confound because valproate itself can alter alpha frequency (Zöllner et al., 2021); drug-naive cohorts are therefore particularly informative. Individual alpha frequency also matters methodologically. If two groups have different alpha frequencies, a fixed analysis frequency samples different positions on their respective spectra. Consequently, a connectivity difference at one frequency can arise from spectral displacement rather than from a difference in the underlying interaction itself (Scally et al., 2018).

We previously applied a wavelet-based phase-synchronization framework to absence seizures (Glaba et al., 2023). Here we extend that framework to the interictal state using the weighted phase-lag index (wPLI), which emphasizes consistent non-zero phase-lagged interactions and reduces sensitivity to instantaneous mixing (Vinck et al., 2011). Because wPLI estimated from a short recording is positive even in the absence of coupling, and the size of that baseline depends on the temporal structure of the signal and therefore need not be equal between groups, we assess participant-specific time-shift baselines with surrogate data rather than assuming them equal. We address four related questions: whether individual alpha frequency differs between drug-naive children with CAE and controls; whether the frontal–posterior balance of wPLI differs between groups; whether a difference in global wPLI survives the same correction; and how the regional contrast depends on evaluation at a common frequency versus individual alpha frequency.

## 2 Materials and methods

### 2.1 Participants

The Ethics Committee of Wrocław University of Science and Technology approved this retrospective analysis. The study included 49 drug-naive patients with CAE (31 girls, 18 boys) and 83 control participants (49 girls, 34 boys) whose routine EEG recordings were reported as within normal limits. The groups did not differ in age (patients 7.16 ± 1.49 years, range 6–12; controls 7.45 ± 1.71 years, range 6–13; *p* = 0.32) or sex distribution (*p* = 0.77). Demographic and clinical characteristics of the study groups are summarized in Supplementary Table S1.

The dataset partially overlaps with cohorts analyzed previously. Of the 49 CAE recordings, 29 came from the previously analyzed Wrocław cohort, whereas 20 were newly included recordings from Katowice (*n* = 10) and Kraków (*n* = 10). Previously analyzed recordings from the Warsaw center were not included. Of the 83 control recordings, 53 were newly included (Katowice, *n* = 25; Kraków, *n* = 20; T. Marciniak Hospital, Wrocław, *n* = 8). CAE was diagnosed by a qualified neurologist based on clinical history, age at seizure onset, clinical assessment, and EEG findings, including recorded absence seizures. In pretreatment video-EEG, 279 seizures were recorded, corresponding to 6 ± 4 seizures per patient with a mean seizure duration of 10 ± 6 s.

### 2.2 EEG recordings and preprocessing

EEG was acquired with an Elmiko Digitrack system (BRAINTRONICS B.V., ISO-1032CE amplifier) using 19 Ag/AgCl electrodes in the international 10–20 montage. Electrode impedance was maintained below 5 kΩ, and the acquisition reference was placed on the forehead. Ten recordings were sampled at 200 Hz and 122 at 250 Hz. All recordings were notch filtered at 50 Hz, and those sampled at 250 Hz were resampled to 200 Hz using MNE-Python (version 1.10.2). All recordings were then re-referenced to the average reference before analysis.

For each participant, the first suitable 30-s eyes-closed resting EEG segment was manually selected by a qualified neurologist, from the beginning of the recording whenever possible. Segments containing ocular, muscle or other artifacts, signs of drowsiness, or interictal epileptiform discharges (IEDs) were excluded. Segment selection was not blinded to diagnosis. Two seconds were discarded from each end of the segment to minimize wavelet edge effects, leaving 26 s (5 200 samples) for the synchronization analyses; all window counts and segment lengths given below for those analyses refer to this 26 s segment. In 38 of 49 patients, the analyzed segment preceded the first recorded seizure. In the remaining 11 patients, the interval from the end of the preceding seizure to the start of the analyzed segment had a median of 54 s (IQR 16–101 s, range 15–258 s).

### 2.3 Individual alpha frequency

IAF was estimated from the full 30-s segment; the wavelet-based synchronization analysis used the central 26 s of the same segment. Power spectral density was calculated separately for seven posterior electrodes (O1, O2, P3, Pz, P4, T5, T6) using the multitaper method. Eight-second windows with 50 % overlap were analyzed with time–bandwidth product *NW* = 3 and five Slepian tapers, and spectra were averaged across windows. The resulting spectral smoothing was approximately 0.75 Hz.

Each channel spectrum was parameterized with specparam (version 2.0.0rc7; Donoghue et al., 2020) over 3–40 Hz using a fixed, knee-free aperiodic model, with peak width limits of 1.5–8 Hz, at most six peaks, a minimum peak height of 0.05 (log_10_ power above the aperiodic fit) and a peak detection threshold of 2 standard deviations of the flattened spectrum. Candidate alpha components had center frequencies between 6 and 14 Hz. Because a broad alpha rhythm is often represented by more than one adjacent fitted component in a short recording, the strongest component was used as an anchor and all in-band components within 2.5 Hz of it were combined. A centroid was then calculated from their fitted center frequencies, weighted by each component's fitted peak height above the aperiodic background. This is a center of gravity suggested by Corcoran et al, who show that a peak estimator is unreliable when the alpha band is not well described by a single component (Corcoran et al., 2018). Our IAF algorithm differs from their implementation in being computed on the parameterized spectrum rather than on a smoothed raw spectrum, so that the aperiodic background does not contribute to the weighting. Channels with model fit *R*^2^ <0.80 were excluded. Participant-level IAF was defined as the median centroid across valid posterior channels and was accepted when at least three of the seven channels yielded a valid estimate. Split-half reliability was assessed by repeating the procedure on alternating multitaper windows and applying the Spearman–Brown correction. Because the 8-s windows overlap by 50 %, the two sets share samples, which can inflate their agreement. This coefficient is therefore reported as an internal-consistency check.

### 2.4 Phase synchronization

The complex Morlet continuous wavelet transform was applied to each channel with center-frequency parameter *f*_*c*_ = 1, so that the wavelet at pseudo-frequency *f* has a temporal standard deviation of 1/*f* and a full width at half maximum of its power response of approximately 0.26*f*; the pseudo-frequency is *f* = *f*_*c*_/(*sΔt*) for scale *s* and sampling interval Δ*t*. The implementation is our own (Glaba et al., 2023).

Synchronization was quantified using the weighted phase-lag index (wPLI; Vinck et al., 2011), which weights each observation of the imaginary cross-spectrum by its magnitude, so that observations close to the real axis contribute little. Writing Xt=Im(Wx(t)Wy*(t)) for the imaginary cross-spectrum of a pair of channels at the selected analysis frequency, the analyzed segment was divided into *M* non-overlapping 1 s windows, and the index was calculated as


$$\begin{array}{l} \gamma =\frac{\left|\sum_{i=1}^{M}\sum_{t\in W_{i}}X_{t}\right|}{\sum_{i=1}^{M}\sum_{t\in W_{i}}|X_{t}|}. \end{array}$$ (1)

Thus, signed contributions were summed across all windows before taking the absolute value, and the denominator was accumulated over the same observations. Because the sums run over the whole segment, repartitioning it into windows of a different length leaves Equation 1 unchanged.

An alternative calculation forms the ratio within each window and averages the resulting values,


$$\begin{array}{l} \gamma_{\text{avg}}=\frac{1}{M}\overset{M}{\underset{i=1}{\sum }}\frac{\left|\sum_{t\in W_{i}}X_{t}\right|}{\sum_{t\in W_{i}}|X_{t}|}. \end{array}$$ (2)

These estimators generally differ. Equation 1 is the absolute value of a weighted mean of the signed window ratios, with weights proportional to each window's summed absolute imaginary cross-spectrum, and permits cancellation between windows with opposite signs. Equation 2 instead takes the absolute value within each window and assigns equal weight to the resulting ratios. Consequently, differences between the estimators can reflect both finite-sample effects and variation in the imaginary cross-spectrum across windows. We refer to Equation 1 as wPLI and to Equation 2 as window-averaged wPLI. We report the former throughout; analyses were repeated with the latter, and the outcomes are reported where the two are compared.

Non-overlapping windows were used to avoid direct sample sharing between the interleaved windows assigned to the two halves of the split-half reliability analysis. Such sharing can inflate reliability estimates. Non-overlap does not, however, ensure statistical independence, because temporal autocorrelation and wavelet smoothing can induce dependence between adjacent windows. To obtain the time course in Supplementary Figure S1, wPLI was also calculated separately within each 1 s window.

Analyses were performed at a single wavelet pseudo-frequency rather than aggregated over a frequency band, so that the frequency dependence of each measure could be examined directly. Each wavelet estimate nevertheless has a finite spectral bandwidth. The fixed analysis frequency was 12 Hz, carried over from our analysis of absence seizures in these recordings (Glaba et al., 2023), which used the same wavelet and pseudo-frequency definition and identified 12 Hz as informative for seizure detection and for characterizing fragmentation of the discharge. Sensitivity to this choice is reported in the Results.

Sample wPLI generally has a positive finite-sample expectation under the null of no coupling. Under stationary sampling with sufficiently weak temporal dependence, the offset of Equation 1 decreases toward zero as the effective sample size increases. Averaging additional fixed-length window estimates, by contrast, does not remove their within-window offset. The null distribution depends on the temporal dependence and distribution of the imaginary cross-spectrum and may therefore differ between groups, even when the number of sampled time points is equal. The surrogate procedure described below was used to assess participant-specific time-shift baselines.

### 2.5 Regional measures

Global synchronization was defined as the mean pairwise wPLI, Equation 1, over all 171 unique electrode pairs. Regional synchronization was defined as the mean wPLI over within-region pairs, computed separately for a frontal set (Fp1, Fp2, F7, F8, F3, F4, Fz) and a posterior set (O1, O2, P3, Pz, P4, T5, T6). Each region contained seven electrodes and 21 unique within-region pairs. T3, T4, C3, C4 and Cz were included in global and node-level analyses but were not assigned to either regional set.

The frontal–posterior balance was summarized by


$$\begin{array}{l} \lambda_{\text{FP}}=\ln\left(\frac{\gamma_{\text{F}}}{\gamma_{\text{P}}}\right), \end{array}$$ (3)

where γ_F_ and γ_P_ denote mean frontal and posterior wPLI, respectively. Negative values indicate posterior predominance, positive values frontal predominance, and zero equal regional means.

For topographic analyses, node strength was defined as the mean wPLI between an electrode and the other 18 electrodes. To examine frequency dependence, all measures were also calculated from 6 to 14 Hz in 0.25 Hz steps.

### 2.6 Finite-sample offset and surrogate correction

Because sample wPLI can be positive in the absence of coupling, participant-specific time-shift baselines were estimated rather than assumed equal between groups. We did not use the debiased estimator of squared wPLI (Vinck et al., 2011), whose independence-based correction does not generally account for temporal dependence between wavelet coefficients; it also targets squared wPLI rather than the statistic analyzed here. Surrogate correction was applied separately to each statistic—global, regional and node-level wPLI, and the regional log-ratio. Corrected values can be negative; for the log-ratio the logarithms were therefore formed before baseline subtraction, as specified below.

For each participant and measure, a surrogate distribution was obtained by recomputing the index after circularly shifting each channel's wavelet coefficients within the analyzed segment (Supplementary Figure S2). A circular shift preserves each channel's coefficient sequence up to a cyclic rotation: its Fourier magnitudes and circular autocorrelation are preserved exactly, while its amplitude envelope is translated. Shifting also changes how features of the coefficient sequence align with the analysis-window boundaries. What the shift alters is the temporal alignment between channels. It therefore removes inter-channel dependence that does not persist across the imposed lag, and leaves any that does; the mean surrogate value is accordingly a participant-specific time-shift baseline rather than the value expected under a general no-coupling null (Schreiber and Schmitz, 2000). Surrogates that instead permute time points or substitute temporally independent noise are unsuitable here, since they reduce the coefficient autocorrelation and so lower the offset along with the coupling.

Because every channel is shifted, the relationship the index reports for a pair depends on the difference between the two lags and not on either lag alone; shifting both channels of a pair equally leaves their relative timing intact. The constraint on the lags was therefore placed on their pairwise circular separation rather than on each channel's own displacement. A minimum pairwise separation of 1 s was imposed, about six times the coherence time of the 12-Hz phase and 3.4 times that of the 6-Hz phase, where the coherence time is the lag at which the modulus of the phase autocorrelation $\left|{\langle e^{i\left[\phi \left(t+\Delta \right)-\phi \left(t\right)\right]}\rangle }_{t}\right|$ first falls to 1/*e* (0.17 and 0.30 s respectively, averaged over channels and participants), and close to the largest separation the 26 s segment can accommodate for 19 channels. Independent draws satisfy this constraint only rarely, so the lag set was constructed rather than sampled by rejection: the segment was divided into 19 gaps, each the 1 s minimum plus a share of the remaining 7 s, the 7 s being partitioned uniformly over the simplex of 19 non-negative shares. Cumulative gap lengths defined the shift positions, and a common offset drawn uniformly over 26 s was applied modulo the segment duration; this rotation made each position marginally uniform around the circle while the positions remained mutually constrained. The positions were then assigned to channels in random order. Because any two positions are separated by at least one gap, the 1 s minimum holds for every one of the 171 pairs regardless of the assignment. The random assignment matters for a different reason: the frontal and posterior electrode sets are contiguous in the montage, and assigning positions in channel order would give within-region pairs systematically smaller separations than between-region pairs, so that the surrogate baseline would acquire a regional structure of its own.

Whether shifts of this size are sufficient is a property of the data rather than of the wavelet, whose temporal support says nothing about how long inter-channel relationships persist. We therefore measured the mean pairwise index as a function of an imposed relative lag, expressed each participant's curve as its excess over that participant's own mean across lags of 2–11.5 s, and averaged the excess over the distribution of pairwise separations that the construction produces. Lags below 0.25 s were excluded from this assessment, because a time shift introduces a carrier-frequency-dependent phase rotation that can convert an instantaneous shared component into a non-zero imaginary cross-spectrum, producing lag dependence unrelated to persistence. This exclusion does not eliminate phase-rotation effects at longer lags, so the curve is interpreted as an empirical lag-sensitivity assessment rather than as a direct measure of the persistence of coupling. The separation-weighted average was calculated over the evaluated separations up to 11.5 s, with the corresponding weights renormalized. Separations between 11.5 and 13 s, comprising approximately one tenth of the surrogate separation distribution, were not evaluated. The outcome is reported in Section 3.3.

One hundred surrogate realizations were generated per participant. For the frequency sweep the same lag realizations were used at every frequency, so that each realization is a complete surrogate spectrum. Each realization was analyzed exactly as the observed data—the same windows, the same construction of the index—and surrogates were generated separately for wPLI and for window-averaged wPLI, since the two constructions have different finite-sample offsets.

For global, regional and node-level wPLI the corrected value was the observed index minus the participant's mean surrogate value,


$$\begin{array}{l} \gamma^{\text{corr}}=\gamma^{\text{obs}}-\frac{1}{Q}\overset{Q}{\underset{q=1}{\sum }}\gamma_{q}, \end{array}$$ (4)

where γ_*q*_ is the index computed on the *q*th of *Q* = 100 realizations. For λ_FP_ the surrogate log-ratio was formed from the surrogate regional values within each realization and the correction applied in the log domain,


$$\begin{array}{l} \lambda_{\text{FP}}^{\text{corr}}=\ln\frac{\gamma_{\text{F}}^{\text{obs}}}{\gamma_{\text{P}}^{\text{obs}}}-\frac{1}{Q}\overset{Q}{\underset{q=1}{\sum }}\ln\frac{\gamma_{\text{F},q}}{\gamma_{\text{P},q}}. \end{array}$$ (5)

The regional values were not corrected before the ratio was formed, because the correction is additive on the wPLI scale whereas λ_FP_ is nonlinear, and corrected regional values can be negative. Nor was the surrogate log-ratio formed from the mean surrogate regional values, since the mean of the logarithms is not the logarithm of the ratio of means; Equation 5 places each realization on the scale of the observed value before averaging. No observed or surrogate regional mean was zero, so no floor value was required.

Subtracting the mean of the surrogate distribution is a baseline subtraction and not a provably unbiased estimator of the underlying coupling, since the offset need be neither additive nor independent of coupling strength. What the corrected quantity supports is a between-group comparison of the excess over each participant's own baseline, which is the use made of it here.

### 2.7 Synchronization at individual alpha frequency

As a sensitivity analysis, observed and surrogate frontal–posterior log-ratios were evaluated at each participant's IAF, using the same participant-specific frequency for both. Baseline subtraction was performed in the log domain as in Equation 5. The patient without an identifiable IAF was excluded from both the IAF and 12 Hz evaluations, so comparisons used the same 48 patients and 83 controls. Values at IAF were obtained by linear interpolation of each participant's observed and mean surrogate log-ratio spectra, evaluated in 0.25 Hz steps over 6–14 Hz, to that participant's IAF. No extrapolation was required.

For participant *i*, the change between frequency choices was defined as


$$\begin{array}{l} d_{i}=\lambda_{\text{FP},i}^{\text{corr}}\left(12\text{ Hz}\right)-\lambda_{\text{FP},i}^{\text{corr}}\left(\text{IA}{\text{F}}_{i}\right). \end{array}$$ (6)

Comparing *d*_*i*_ between groups directly assesses whether the regional group contrast differs between the two frequency choices, while preserving the pairing of measurements within participants.

### 2.8 Statistical analysis

Group comparisons of continuous measures used a two-sided studentized permutation test. The statistic permuted was the Welch *t*, computed directly from the two samples without assuming equal variances, rather than the raw difference in means; under unequal variances the raw difference is not pivotal, whereas the Welch statistic is asymptotically so, and the permutation test is then asymptotically valid under unequal variances. Group labels were permuted 10,000 times, and the observed statistic was included in the null distribution so that the reported *p* is (1+*m*)/(1+10000), where *m* is the number of permuted statistics at least as extreme as the observed one. Confidence intervals on group differences are normal-theory Wald intervals, the difference in means ±1.96 times its Welch standard error; they are reported alongside permutation *p* values and are not themselves permutation-based. Effect sizes are Hedges' *g*, the difference in means divided by the pooled standard deviation and multiplied by the small-sample correction *J* = 1 − 3/[4(*n*_1_+*n*_2_−2)−1]. No correction for multiplicity was applied across the scalar outcomes (IAF, global and regional wPLI, λ_FP_, and their surrogate and corrected versions); reported *p* values are unadjusted.

The corrected log-ratios at 12 Hz and IAF and the within-participant changes *d*_*i*_ (Equation 6) were compared between groups using the same two-sided Welch-studentized permutation procedure, with 10 000 participant-label permutations. Confidence intervals used the Welch standard error of the corresponding participant-level quantity. These sensitivity comparisons were exploratory and their *p*-values were not adjusted for multiplicity.

The difference in IAF was additionally adjusted for age by ordinary least squares regression of IAF on group and age, the group coefficient being reported with its Wald interval and parametric *p* value; this is the one comparison in the paper not tested by permutation. The regional posterior–frontal difference was formed within each participant before comparison, so that both regional values from the same recording enter the same quantity; the comparison of that quantity between groups is a between-group permutation test as above, not a paired test.

Split-half reliability was assessed by computing each index on two halves of the window sequence and correlating the halves across participants. Two splits were used: interleaved, assigning odd- and even-numbered windows to the two halves so that both sample the whole recording; and contiguous, using the first and second halves of the sequence, so that a difference between halves includes change over the recording as well as measurement noise. Agreement was quantified by Pearson's *r* and by the intraclass correlation for absolute agreement under a two-way random-effects model with single measurement, ICC(2,1), and both were projected to the full recording length by the Spearman–Brown formula 2*r*/(1+*r*).

For the electrode-wise topography, multiplicity across the 19 electrodes was addressed using a maximum-statistic permutation procedure, which controls the family-wise error rate under the complete null, based on the Welch statistic—the same statistic used for the scalar outcomes. Group labels were randomly permuted 10 000 times at participant level, applying the same permutation across all electrodes. For each permutation, the Welch statistic, including its variance estimate, was recomputed at every electrode, and the largest absolute statistic was retained. The adjusted *p*-value for electrode *j* was *p*_adj, *j*_ = (1+*m*_*j*_)/(1+10000), where *m*_*j*_ is the number of permutation maxima at least as large as the absolute observed Welch statistic at electrode *j*. Studentization expresses each mean difference relative to its estimated standard error, reducing the influence of differences in variability across electrodes on the maximum statistic. Multiplicity correction was performed separately for the observed and surrogate-corrected analyses, each across all 19 electrodes.

Discrimination between groups by the corrected λ_FP_ is summarized by the area under the ROC curve, obtained from the Mann–Whitney *U* statistic, and by the proportion of patients above the control median. No confidence interval is reported for the AUC; it is given as a descriptive summary of separation in this sample.

The frequency dependence of the group difference was examined by repeating the comparison at each of the 33 frequencies of the sweep. No correction for multiple comparisons was applied across frequencies; the sweep is reported as a description of where the effect lies and how it varies, not as a set of independent tests, and adjacent frequencies are not independent in any case, the wavelet integrating approximately ±1.3 Hz at 10 Hz.

Analyses were performed in Python 3.11.14 using NumPy 2.3.3, SciPy 1.16.2, statsmodels 0.14.5, MNE-Python 1.10.2 and specparam 2.0.0rc7.

## 3 Results

### 3.1 Alpha frequency and synchronization spectra

An IAF could be determined in 48 of 49 patients and in all 83 controls. Split-half reliability of the estimate was high in both groups (Spearman–Brown corrected *r* = 0.96 in patients, 0.97 in controls).

Patients had a lower IAF than controls (8.80 ± 0.94 versus 9.42 ± 0.99 Hz; difference −0.62 Hz, 95 % CI [−0.96, −0.27], *p* = 0.002, Hedges' *g* = −0.63; Figure 1A). Because individual alpha frequency increases with age over this range, the comparison was repeated with age as a covariate; the adjusted difference was essentially unchanged (−0.59 Hz, 95 % CI [−0.94, −0.24], *p* = 0.001). Patients were slightly younger than controls, and since alpha frequency rises with age over this range the age difference acts in the same direction as the group effect; adjustment accordingly reduced the estimate slightly rather than removing it.

**Figure 1:**
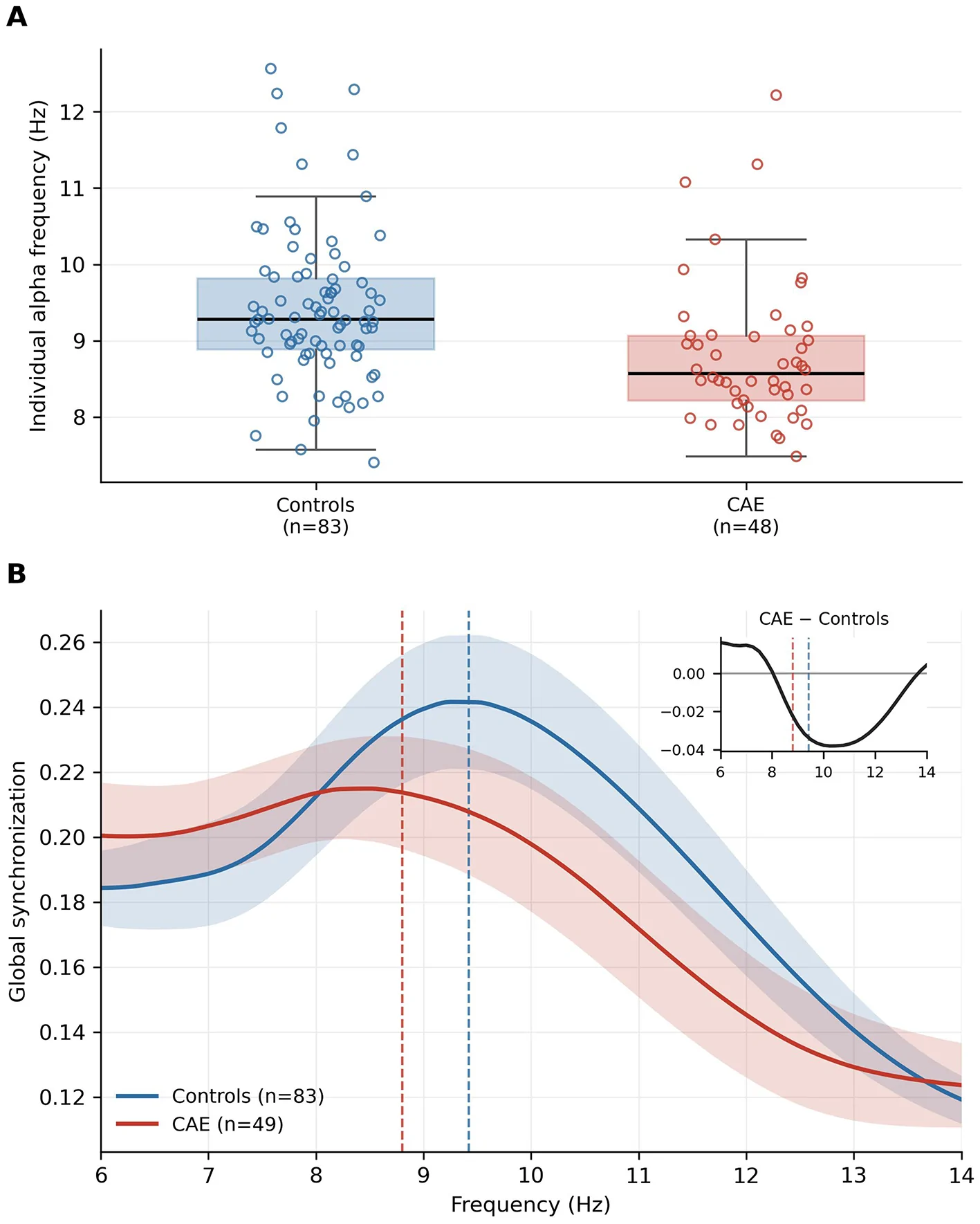
Alpha frequency and phase synchronization. **(A)** Individual alpha frequency (IAF) for each participant, estimated by spectral parameterization of each of seven posterior electrodes' multitaper spectra, the participant value being the median across channels. Boxes give the median and interquartile range; whiskers extend to 1.5 × the interquartile range. The distributions overlap substantially: their standard deviations, 0.94 and 0.99 Hz, exceed the 0.62 Hz separation between their means. **(B)** Global wPLI against analysis frequency, averaged over all electrode pairs. Dashed lines mark each group's mean IAF. Both curves reach a maximum within 0.3 Hz of that line—9.25 Hz in controls, 8.50 Hz in patients. *Inset:* the group difference, which changes sign near 8.1 Hz, is maximal at 10.25 Hz and returns to zero near 13.8 Hz. Shaded bands are ±1 standard error of the group mean. They are computed within each group, so their overlap is not a test of the difference between groups; that difference is shown in the inset. Adjacent frequencies are not independent, the wavelet integrating approximately ±1.3 Hz at 10 Hz. *n* = 49 patients, *n* = 83 controls; IAF could not be determined in one patient.

The observed global wPLI spectra and their between-group difference varied with analysis frequency (Figure 1B). At 12 Hz, global wPLI was lower in patients than in controls (difference −0.028, 95 % CI [−0.054, −0.003], *p* = 0.031, *g* = −0.38). The sensitivity of the regional contrast to frequency choice was assessed by comparing evaluation at 12 Hz with evaluation at each participant's IAF, as reported below.

Global pooled wPLI showed within-recording reproducibility at 12 Hz. Across both cohorts, the interleaved split yielded Pearson's *r* = 0.78 and an absolute-agreement intraclass correlation of 0.78; the contiguous split yielded *r* = 0.64. Spearman–Brown projections of full-recording reliability were 0.88 and 0.78, respectively. Interleaved correlations were 0.77 in controls and 0.82 in patients. These estimates describe within-recording agreement and can reflect both persistent coupling and a stable participant-specific baseline.

### 3.2 Spatial distribution of synchronization

The observed node-strength difference was negative across the scalp, with the largest reduction occipitally (Figure 2A). Thus, the lower global mean was accompanied by a spatially uneven reduction. The pre-specified frontal–posterior comparison below quantifies regional balance using within-region pairs. Node strength, which averages connections from each electrode to all other electrodes, and within-region wPLI describe related but distinct aspects of this spatial pattern.

**Figure 2:**
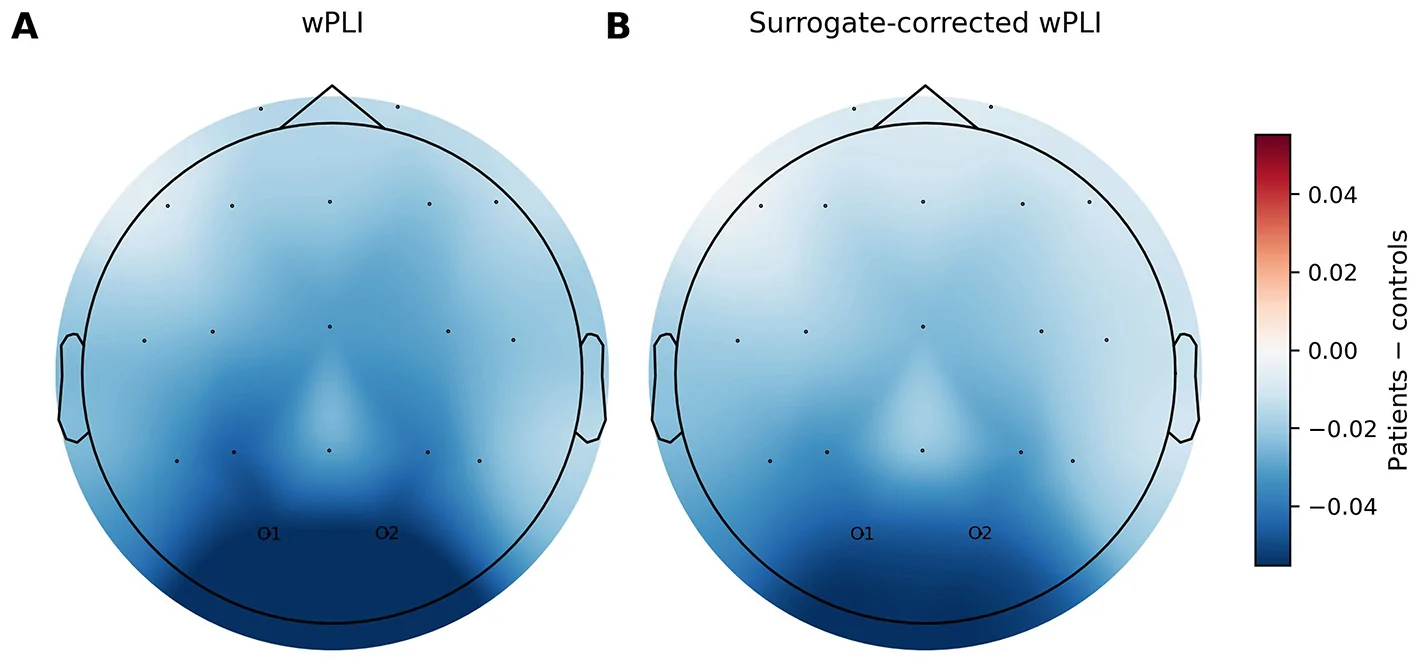
Node strength at 12 Hz, before and after surrogate correction. **(A)** Group difference in node strength, the mean wPLI from each electrode to all others. **(B)** The same difference after subtracting each participant's mean surrogate value. Both panels share a color scale; electrodes are named where the difference survives max-statistic correction across the 19 electrodes. The reduction is graded with an occipital maximum and is negative at every electrode. After correction O1 and O2 survive (*p*_adj_ = 0.012 and 0.043), with P3, T5 and P4 next in order; the occipital reduction is 55–57 % of the control value. *n* = 49 patients, *n* = 83 controls.

### 3.3 Frontal–posterior balance and circular-shift comparison

At 12 Hz, posterior wPLI exceeded frontal wPLI in controls (0.202 versus 0.139), whereas the patient means were nearly equal (0.149 versus 0.147; Table 1).

**Table 1:** Regional wPLI and the frontal–posterior log-ratio at 12 Hz, observed, surrogate and surrogate-corrected.

	Controls	Patients	Difference	95 % CI	*p*	*g*
Observed						
Frontal wPLI	+0.139	+0.147	+0.009	[−0.011, +0.029]	0.397	+0.16
Posterior wPLI	+0.202	+0.149	−0.053	[−0.084, −0.022]	0.001	−0.58
F–P log-ratio	−0.352	+0.016	+0.368	[+0.217, +0.519]	<0.001	+0.88
Surrogate						
Frontal wPLI	+0.108	+0.103	−0.005	[−0.013, +0.003]	0.207	−0.21
Posterior wPLI	+0.112	+0.102	−0.009	[−0.016, −0.003]	0.002	−0.47
F–P log-ratio	−0.036	+0.000	+0.036	[+0.006, +0.067]	0.019	+0.39
Surrogate-corrected						
Frontal wPLI	+0.030	+0.044	+0.014	[−0.006, +0.034]	0.176	+0.25
Posterior wPLI	+0.090	+0.046	−0.044	[−0.072, −0.016]	0.003	−0.55
F–P log-ratio	−0.316	+0.015	+0.332	[+0.181, +0.483]	<0.001	+0.79

Values are group means; differences are patient minus control with 95 % normal-theory confidence intervals, two-sided studentized permutation *p*-values and Hedges' *g*.

Observed log-ratios were calculated within participants. Surrogate log-ratios were calculated within each participant and realization before averaging, and corrected log-ratios were obtained by subtraction in the log domain (Equation 5). The log-ratio entries therefore cannot be reconstructed from the group-mean regional wPLI values. Differences were calculated before rounding. *n* = 49 patients, *n* = 83 controls.

The mean frontal–posterior log-ratio was −0.352 in controls and +0.016 in patients, giving a between-group difference of +0.368, 95 % CI [+0.217, +0.519], *p* < 0.001, *g* = +0.88. Observed posterior wPLI was lower in patients (−0.053, 95 % CI [−0.084, −0.022], *p* = 0.001, *g* = −0.58); the frontal difference was +0.009 (95 % CI [−0.011, +0.029], *p* = 0.397, *g* = +0.16).

We next examined whether the observed pattern was reproduced after circularly shifting each participant's channel coefficients. The lag-sensitivity assessment showed a small mean excess relative to the participant-specific long-lag reference over the evaluated surrogate separations: +0.0003 ± 0.0001 at 12 Hz (mean ± SEM across 132 participants; 2.7 % of the zero-lag excess) and −0.0001 ± 0.0001 at 6 Hz (Supplementary Figure S3). These weighted estimates cover separations up to 11.5 s, approximately 90 % of the surrogate separation distribution, with weights renormalized. They assess the chosen shifts relative to a long-lag reference and do not exclude dependence persisting at those reference lags. The surrogate mean was therefore treated as a time-shift baseline rather than as an estimate of finite-sample bias alone.

At 12 Hz, circular shifting largely removed posterior predominance in controls: the mean log-ratio changed from −0.352 in the observed data to −0.036 in the surrogates. The patient mean remained near zero in both cases (Table 1). Absolute wPLI decreased in both regions and both cohorts, but the reduction was larger posteriorly than frontally in controls and similar across regions in patients. Control frontal and posterior means changed from 0.139 and 0.202 to 0.108 and 0.112, respectively; patient means changed from 0.147 and 0.149 to 0.103 and 0.102. The similarity of the patient log-ratios therefore did not imply unchanged synchronization.

Figures 3A, B show the observed, surrogate and observed-minus-surrogate log-ratios across frequency in each cohort. In controls, the pronounced negative observed log-ratio across upper alpha was substantially attenuated in the surrogates, while the observed-minus-surrogate curve remained negative. In patients, the observed and observed-minus-surrogate means were near zero at 12 Hz, but were negative at lower frequencies, particularly around 8–10 Hz. Thus, the near-zero patient mean at the primary analysis frequency does not describe the entire frequency range. The shaded intervals summarize uncertainty in each group mean at each frequency; between-group inference is based on the participant-level comparisons reported below.

**Figure 3:**
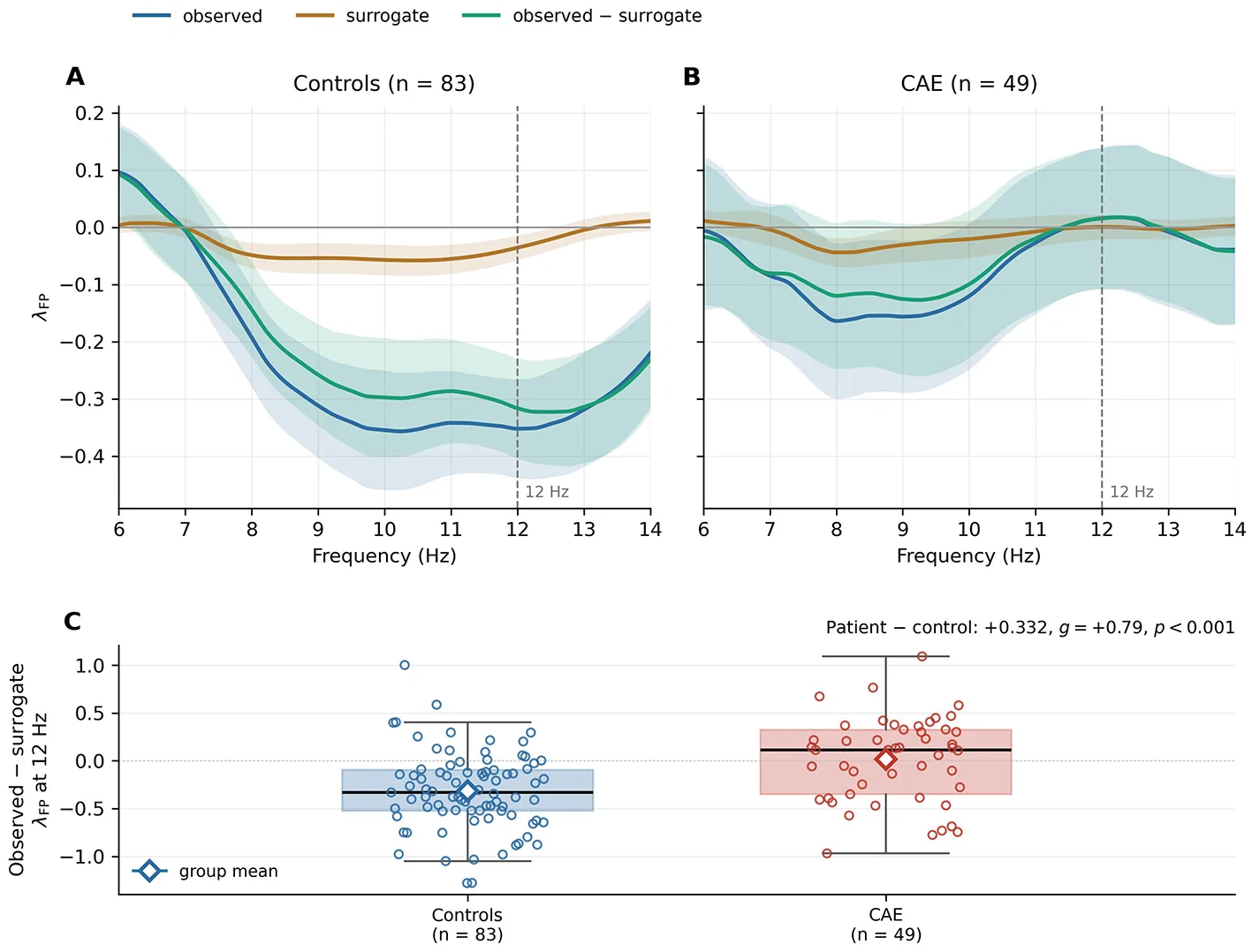
Frontal–posterior log-ratio across frequency and at the analysis frequency. Group means of observed λ_FP_ (blue), circular-shift surrogate λ_FP_ (orange), and observed minus surrogate values (green), in **(A)** controls and **(B)** patients. Surrogate log-ratios were formed within each realization and averaged within each participant before group averaging (Equation 5). Negative observed or surrogate values indicate posterior predominance. The green curve represents subtraction in the log domain, not the log-ratio of baseline-subtracted regional wPLI. Shaded bands are pointwise 95 % normal-theory confidence intervals, calculated as the group mean ±1.96 standard errors across participants at each frequency; for the green curve the standard errors were calculated from participant-level observed-minus-surrogate values, accounting for their pairing. These intervals are not simultaneous confidence bands across frequencies. Panels A and B share axis limits; dashed vertical lines mark the 12 Hz analysis frequency. **(C)** The participant-level values underlying the green curves at 12 Hz. Boxes give the median and interquartile range, whiskers extend to the most extreme observations within 1.5 × the interquartile range of the box edges, and open circles show individual participants. The patient-minus-control mean difference was +0.332, 95 % CI [+0.181, +0.483], *p* < 0.001, Hedges' *g* = +0.79. *n* = 83 controls and *n* = 49 patients.

The surrogate log-ratio retained a small between-group difference (+0.036, 95 % CI [+0.006, +0.067], *p* = 0.019, *g* = +0.39). Subtracting each participant's surrogate mean log-ratio left a difference of +0.332, 95 % CI [+0.181, +0.483], *p* < 0.001, *g* = +0.79 (Figure 3C). The participant-level values in panel C are those underlying the green curves in panels A and B at 12 Hz. This comparison tests the between-group difference in observed-minus-surrogate changes. Approximately 90 % of the observed log-ratio difference remained, calculated as the corrected difference divided by the observed difference.

After baseline subtraction, posterior wPLI was lower in patients (−0.044, 95 % CI [−0.072, −0.016], *p* = 0.003, *g* = −0.55), whereas the frontal difference was +0.014 (95 % CI [−0.006, +0.034], *p* = 0.176, *g* = +0.25). A direct between-group comparison of each participant's corrected posterior-minus-frontal wPLI gave a difference of −0.058, 95 % CI [−0.085, −0.031], *p* < 0.001, *g* = −0.74. This supported a regional contrast without relying on a comparison of the significance of the two separate regional tests.

The corrected node-strength difference also remained most pronounced occipitally (Figure 2B). O1 and O2 retained group differences under the Welch maximum-statistic correction across 19 electrodes (*p*_adj_ = 0.012 and 0.043, respectively), with P3, T5 and P4 next in order. The corrected reduction at the occipital electrodes was 55–57 % of the control value.

For global wPLI, the corrected group difference did not reach statistical significance (−0.021, 95 % CI [−0.044, +0.001], *p* = 0.069). The pooled surrogate mean in controls was 0.109, compared with an observed mean of 0.174, representing 63 % of the observed index. The interval on the corrected comparison was compatible with both no difference and a reduction in patients. Group separation for the corrected regional log-ratio was partial: the AUC was 0.72, and 73 % of patients lay above the control median.

### 3.4 Frequency choice and sensitivity analyses

IAF ranged from 7.41 to 12.56 Hz among the 131 participants with an identifiable estimate. In this common sample, the patient-minus-control difference in observed-minus-surrogate λ_FP_ was +0.316 at 12 Hz (95 % CI [+0.166, +0.466], *p* = 0.0002, *g* = +0.76) and +0.124 at IAF (95 % CI [−0.038, +0.287], *p* = 0.139, *g* = +0.28; Figures 4A, B). The 12 Hz estimate differs slightly from the full-sample result because the patient without an IAF estimate was excluded from both evaluations.

**Figure 4:**
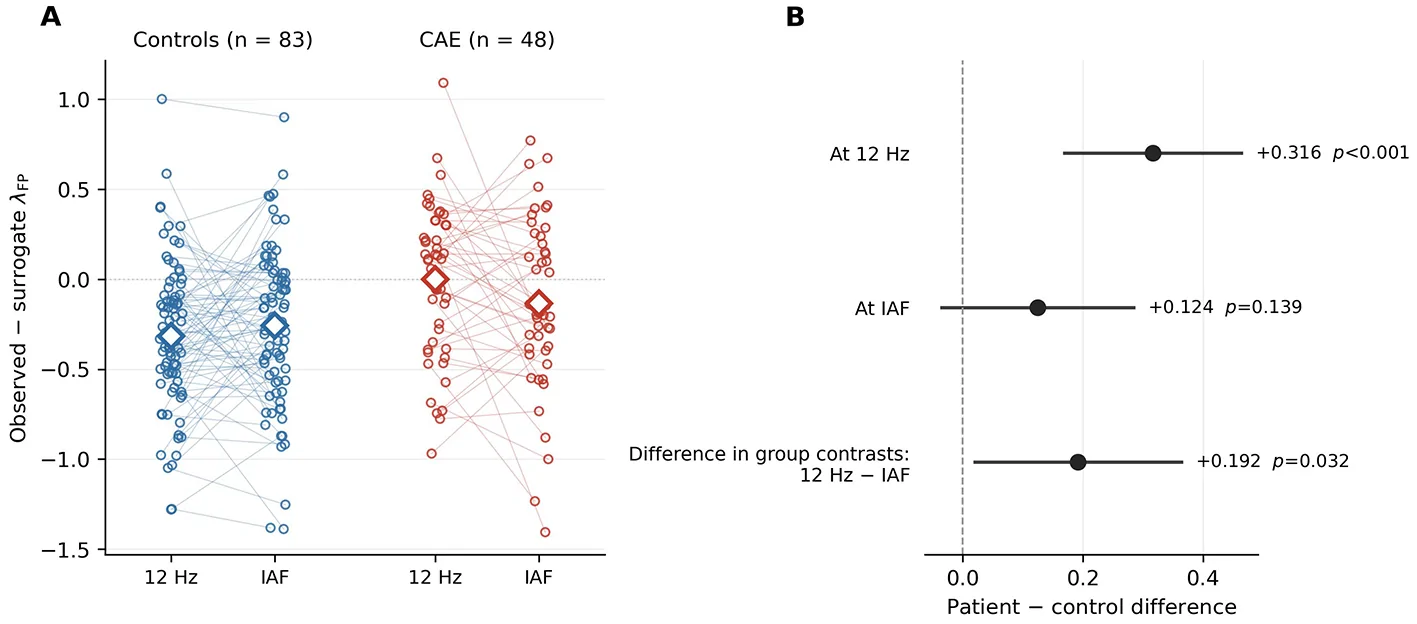
Regional group contrasts at a fixed frequency and at individual alpha frequency. **(A)** Observed-minus-surrogate λ_FP_ at 12 Hz and at each participant's individual alpha frequency (IAF). Open circles represent participants, connecting lines join their paired values, and open diamonds mark group means. The patient without an IAF estimate is excluded from both evaluations, leaving the same 131 participants in each comparison. **(B)** Patient-minus-control mean differences at 12 Hz and at IAF, and the difference between these group contrasts. The third estimate was obtained by calculating the 12 Hz-minus-IAF difference within each participant and comparing these differences between groups. It directly assesses whether the group contrast differs between frequency choices; significance in one comparison and nonsignificance in the other would not establish this. Points show estimates and horizontal bars show 95 % normal-theory Wald confidence intervals. Two-sided *p*-values are from studentized permutation tests and are unadjusted for multiplicity. *n* = 83 controls and *n* = 48 patients.

The direct comparison of the within-participant 12 Hz-minus-IAF changes yielded a between-group difference of +0.192 (95 % CI [+0.017, +0.367], unadjusted *p* = 0.032, *g* = +0.39; Figure 4B). Thus, the regional group contrast was larger at 12 Hz than at IAF. The interval for the IAF contrast included zero and positive differences, so this result does not establish equivalence between groups at IAF.

Repeating the log-ratio analysis with window-averaged wPLI gave a smaller corrected difference in the same direction (+0.050, 95 % CI [+0.018, +0.082], *p* = 0.002, *g* = +0.54). For global wPLI, this construction also gave an unresolved corrected difference (−0.002, *p* = 0.59). Its surrogate mean in controls was 0.469 against an observed mean of 0.482, or 97 % of the observed index.

Excluding the 11 patients whose analyzed segment followed a recorded seizure left the corrected log-ratio difference similar (+0.339, 95 % CI [+0.182, +0.496], *g* = +0.82, *n* = 38 patients). In the descriptive frequency sweep, the corrected log-ratio difference was significant in point-wise, unadjusted comparisons from 9.75 to 14 Hz, with *g* = +0.41 at 10 Hz, a maximum of +0.79 near 12.25 Hz, and +0.43 at 14 Hz.

To assess whether the regional finding was consistent across recording subsets, the comparison of surrogate-corrected λ_FP_ was repeated separately in recordings from Wrocław (29 patients, 38 controls) and from the other centers (20 patients, 45 controls). The patient-minus-control difference was positive in both subsets: +0.258 (95 % CI [+0.074, +0.442], *g* = +0.67) in Wrocław and +0.408 (95 % CI [+0.150, +0.666], *g* = +0.87) in the other centers. Within each group, the corrected index showed no statistically significant difference between the two subsets (controls *p* = 0.28; patients *p* = 0.69). These results indicate that the regional finding was not confined to either recording subset, although this stratification does not exclude confounding by individual recording center.

## 4 Discussion

Drug-naive children with childhood absence epilepsy (CAE) had a slower resting alpha rhythm and reduced posterior predominance of alpha-band phase synchronization compared with controls of similar age. Controls showed greater posterior than frontal synchronization at 12 Hz, whereas the patient mean frontal–posterior log-ratio was near zero at that frequency. Circular shifting largely removed the controls' posterior predominance, while the patient mean balance remained near zero despite reductions in absolute wPLI. The between-group difference in observed-minus-surrogate log-ratios was significant, indicating that the cohorts differed in how their regional balance responded to altered channel alignment.

The regional contrast depended on frequency choice. In the same 131 participants, the corrected group difference was larger at 12 Hz than at individual alpha frequency, as established by a direct comparison of within-participant changes. A group difference at IAF was not established. The global synchronization difference did not remain statistically significant after baseline subtraction under either construction of wPLI.

### 4.1 What the circular-shift comparison shows

At 12 Hz, the contrast between cohorts lies in the regional balance of the reduction produced by shifting, not in whether synchronization fell. It fell in both regions and both cohorts; in controls it fell further posteriorly, so that a marked posterior predominance in the observed data was largely absent in the surrogates, whereas in patients the two regions fell together and the balance was near zero in both conditions. Figure 3 brings these two aspects together: panels A and B show each cohort's frequency-dependent response to shifting, and panel C shows the between-group comparison of the observed-minus-surrogate values at 12 Hz. This comparison is the same statistical contrast reported as the surrogate-corrected group difference, rather than an additional test.

The patient spectra also qualify the interpretation. Their mean regional balance was near zero at 12 Hz, but showed posterior predominance at lower frequencies. The finding therefore concerns altered frequency-dependent regional organization, rather than an absence of posterior predominance throughout the alpha range.

Two features of the shift make that comparison informative. It retains each channel's coefficient sequence up to a cyclic rotation, including its Fourier magnitudes and circular autocorrelation. It also preserves each channel's amplitude distribution and shifts its amplitude envelope intact, while altering the alignment of both amplitudes and phases between channels. Random assignment of the constrained shifts avoids a systematic association between electrode region and shift separation; it does not force the regional surrogate means to be equal. Their similarity at 12 Hz (approximately 0.10 both frontally and posteriorly in each cohort) was an empirical observation. The controls' posterior predominance was therefore not reproduced when the single-channel properties were retained under the sampled shifts. This argues against those properties being sufficient to explain the observed regional contrast, without attributing the contrast to physiological coupling.

The surrogate log-ratio nonetheless differed between groups, so the baseline was not equal across cohorts and assuming it equal would have obscured a component of the observed contrast that was also present after shifting. Approximately 90 % of the observed group difference remained after subtraction, but that ratio of point estimates does not partition the finding into estimator bias and physiological coupling: circular shifts can retain persistent dependence, and subtraction need not remove the bias of the observed estimator.

The distinction matters most for global synchronization, where the surrogate baseline was approximately 63 % of the observed index in controls under pooling and 97 % under window averaging. The observed group difference was significant under both constructions and the baseline-relative difference under neither, with an interval leaving both zero and a reduction in patients compatible with the data. That result is unresolved rather than null.

The regional contrast, by contrast, held under both constructions, across 9.75–14 Hz in the descriptive sweep, in the 38 patients whose segment preceded the first recorded seizure, and in the electrode-wise analysis, where the two occipital electrodes retained group differences after correction across the montage.

### 4.2 Interpretation

The lower alpha frequency is consistent with previous reports of a shift of alpha activity toward lower frequencies in epilepsy (Abela et al., 2019). The drug-naive cohort excludes prior antiseizure medication as an explanation, which is relevant because valproate can alter alpha frequency (Zöllner et al., 2021), and restricting the cohort to CAE avoids combining syndromes. Alpha frequency was estimated from fitted periodic components after accounting for the aperiodic background rather than from a ratio of band powers. The finding therefore extends observations of alpha slowing to a drug-naive CAE cohort using a measure of periodic alpha activity.

Reduced posterior predominance in phase-lagged synchronization is the principal regional finding. It converges with the altered anterior–posterior organization reported by Rotondi et al. (2016), who used partial directed coherence in eleven drug-naive children with CAE and found that the anterior–posterior gradient of outgoing connection density present in controls was absent in patients, with the strongest differences in the alpha band. Partial directed coherence and within-region wPLI characterize different aspects of connectivity, so the findings are not equivalent; both nonetheless support altered anterior–posterior organization of resting activity in CAE.

The IAF analysis directly qualifies the regional finding (Figure 4). In the common sample, the corrected group contrast was +0.316 at 12 Hz and +0.124 at IAF; the difference between these contrasts was +0.192 (unadjusted *p* = 0.032). This is evidence that the group contrast depends on frequency choice, rather than an inference from significance at one frequency and nonsignificance at the other. It supports altered regional organization at a common upper-alpha frequency, but does not establish a comparable difference at each participant's own alpha frequency. Nor does it establish equivalence at IAF or show that alpha slowing alone causes the regional contrast.

The physiological basis of the regional finding remains uncertain. Scalp-level phase-lag measures do not uniquely identify interactions between underlying neural sources; source mixing, common inputs and referencing can influence their interpretation, and circular shifting does not resolve these ambiguities. The supported conclusion is that the frontal–posterior balance of scalp-level phase-lag consistency differs between groups and remains different relative to participant-specific time-shift baselines. Whether this corresponds to altered physiological coupling between particular brain regions requires complementary analyses.

### 4.3 Limitations

The recordings are short. Thirty-second segments were selected, with 26 s retained for synchronization analysis, and even under pooling the surrogate baseline was nearly two thirds of the observed global index in controls. Longer recordings could reduce the finite-sample component if they provide additional effectively independent observations, but would not eliminate the time-shift baseline.

We carried over the 12 Hz analysis frequency from earlier work (Glaba et al., 2023) because we found it best for seizure detection and for characterizing discharge fragmentation. Herein, the largest regional difference occurred near 12.25 Hz. The IAF comparison was exploratory, and its direct test of the change in group contrast was unadjusted for multiplicity. IAF was itself estimated from a short recording, and the alpha center frequency varied across the seven posterior channels within participants by more than the group difference in IAF; evaluating synchronization at a single per-participant frequency therefore inherits that variability.

The control participants were children referred for routine EEG whose recordings were reported as normal, rather than a population sample, and the groups were compared for age and sex rather than matched. Eleven patients' segments followed a recorded seizure by a median of 54 s; excluding them left the regional finding evident. The findings apply specifically to eyes-closed resting EEG and cannot be assumed to generalize to eyes-open recordings or other behavioral states. Although segments with signs of drowsiness were excluded, subtle differences in vigilance and alpha signal characteristics cannot be fully excluded as contributing factors. The 19-channel montage limits spatial resolution, and the frontal–posterior partition, though pre-specified and balanced, summarizes the spatial pattern coarsely. The design was cross-sectional, and the measures were not related to cognitive performance, seizure burden or clinical outcome.

Split-half reliability supports consistent ranking of participants within a recording but does not establish stability between sessions or clinical utility; the AUC of 0.72 indicates partial separation in this sample and has not been validated independently. The present findings characterize interictal alpha organization, and their diagnostic value remains to be established.

Finally, the surrogate baseline is specific to the chosen transformation and lag distribution. Phase relationships persisting across the sampled shifts contribute to it, and the lag-sensitivity analysis assesses the selected separations relative to a long-lag reference without excluding dependence that also persists at those reference lags. Corrected values are therefore relative to the time-shift baseline, not to a general absence of coupling.

### 4.4 Conclusions

Drug-naive children with CAE showed a slower resting alpha rhythm and reduced posterior predominance of alpha-band phase synchronization. At 12 Hz, circular shifting largely removed posterior predominance in controls while the patient regional balance remained near zero, and the between-group difference in observed-minus-surrogate log-ratios shows that the cohorts differed in how that balance responded to altered channel alignment. The regional contrast at 12 Hz held under both estimator constructions and after exclusion of post-seizure segments. It was smaller when evaluated at each participant's IAF, where a group difference was not established. Frequency choice is therefore integral to the interpretation of the regional finding. The global difference relative to the surrogate baseline remained unresolved.

The surrogates therefore did more than adjust a baseline: they identified which feature of the observed regional organization is not reproduced when channel alignment is changed and the individual coefficient sequences retained. This supports a difference in scalp-level regional organization relative to the selected shifts, while leaving its physiological basis, and the contribution of persistent dependence to the baseline, open.

## Data Availability

The raw data supporting the conclusions of this article will be made available by the authors, without undue reservation.
