# Supplementary material for: Altered interictal alpha-band organization in childhood absence epilepsy
Source: Front Neuroinform. 2026 Oct 23;20:1875377. doi: 10.3389/fninf.2026.1875377 (PMC13739694; doi:10.3389/fninf.2026.1875377)
Supplement: Supplementary file 1 [file Data_Sheet_1.pdf]

Supplementary Material

1 SUPPLEMENTARY DATA

Table S1. Demographic and clinical characteristics of the study groups. The CAE group comprised drug-naïve children evaluated during the pretreatment EEG examination. Control participants underwent EEG for non-epileptic clinical indications and had recordings evaluated as being within normal limits.

Characteristic	CAE patients	Controls
Participants, n	49	83
Age, years, mean $\pm$ SD	7.16 ± 1.49	7.45 ± 1.71
Age range, years	6–12	6–13
Female / male, n	31 / 18	49 / 34
EEG indication	Diagnostic evaluation of suspected CAE	Non-epileptic clinical indications
Treatment status at baseline	Drug-naïve	–
Pretreatment seizures recorded, total n	279	–
Seizures per patient, mean $\pm$ SD	6 ± 4	–
Seizure duration, s, mean $\pm$ SD	10 ± 6	–

2 SUPPLEMENTARY FIGURES

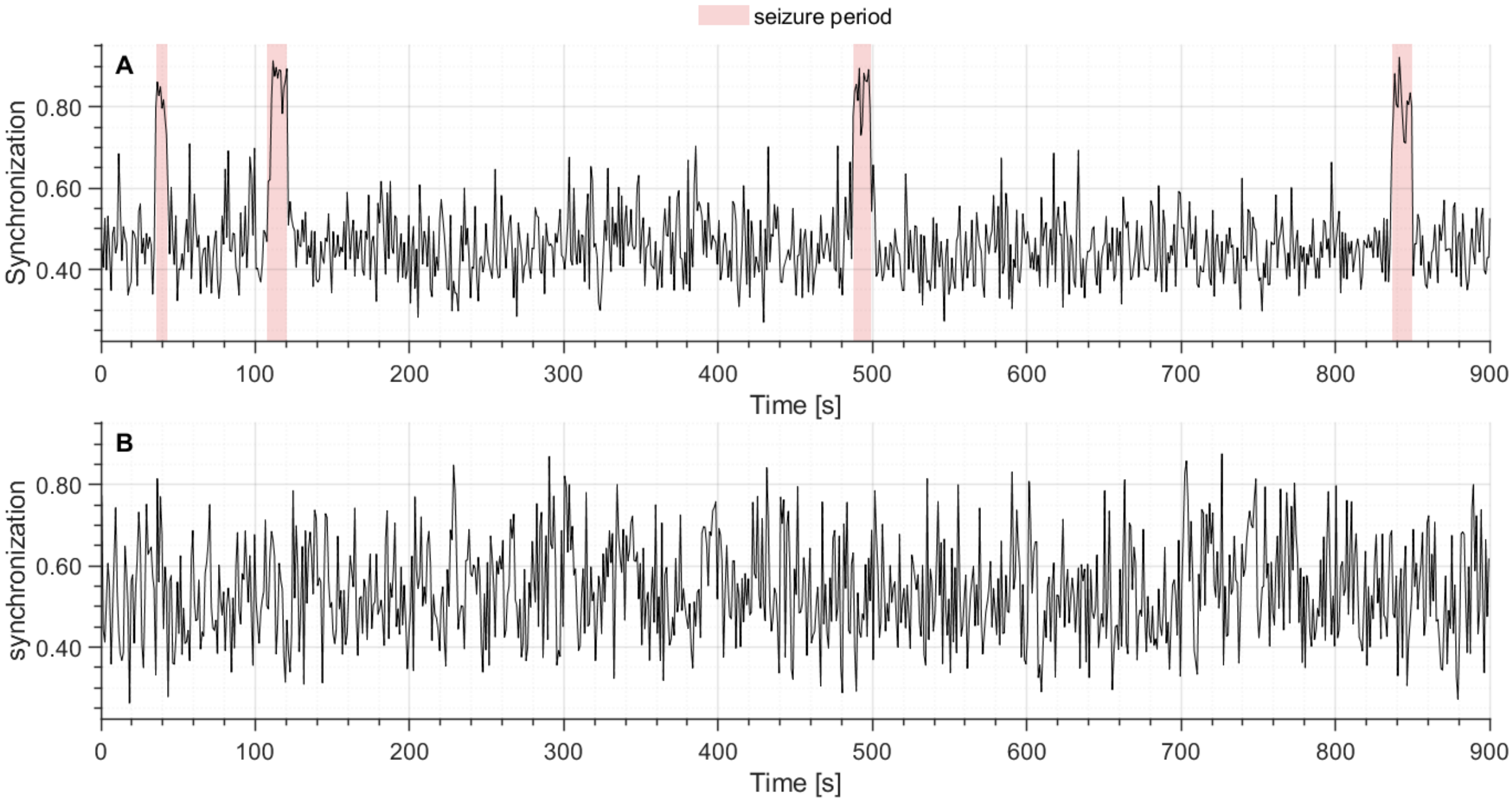

Figure S1: The plots show the temporal evolution of global synchronization, quantified using the weighted phase-lag index (wPLI) at a fixed frequency of 12 Hz. wPLI values were calculated in consecutive, non-overlapping 1-s windows. Panel A shows a representative patient with childhood absence epilepsy, whereas panel B shows a representative control participant. Red-shaded areas indicate absence seizures. These examples illustrate that absence seizures are accompanied by marked increases in synchronization. Interictal synchronization is variable and can transiently reach levels observed during seizures.

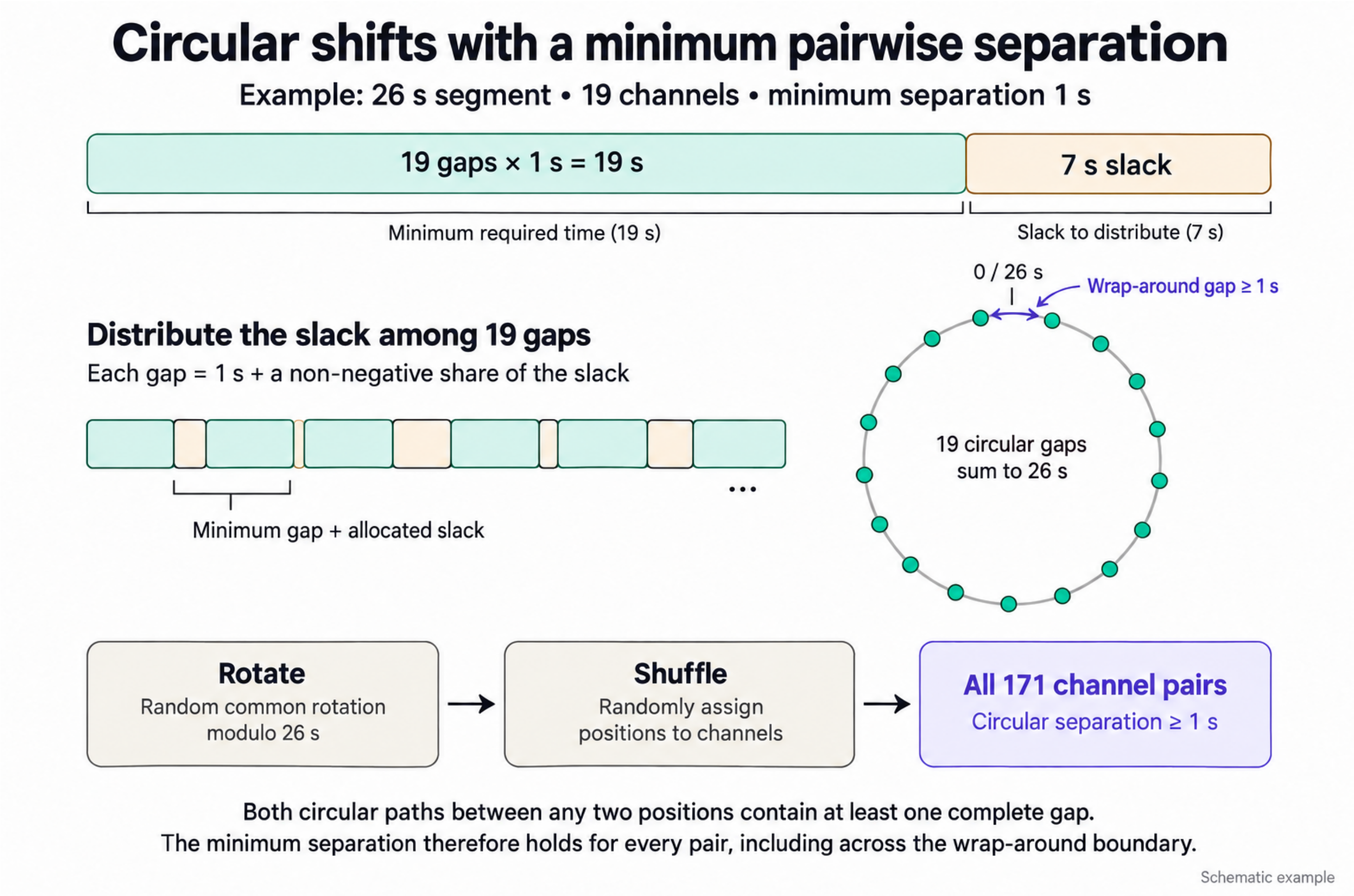

Figure S2: **Construction of circular shifts with a minimum pairwise separation.** For 19 channels and a 26 s segment, imposing a minimum circular separation of 1 s requires 19 circular gaps totalling at least 19 s. The remaining 7 s is partitioned uniformly over the simplex of 19 non-negative shares. Each gap comprises 1 s plus its allocated share. Cumulative gap lengths define 19 shift positions on the circle. A common rotation, sampled uniformly over the segment duration, is applied modulo 26 s, and the positions are randomly assigned to channels. Every circular gap, including the wrap-around gap, is at least 1 s; consequently, the shortest circular separation is at least 1 s for all 171 channel pairs. Random rotation avoids a preferred time origin, while random assignment prevents systematic association between channel order and shift position.

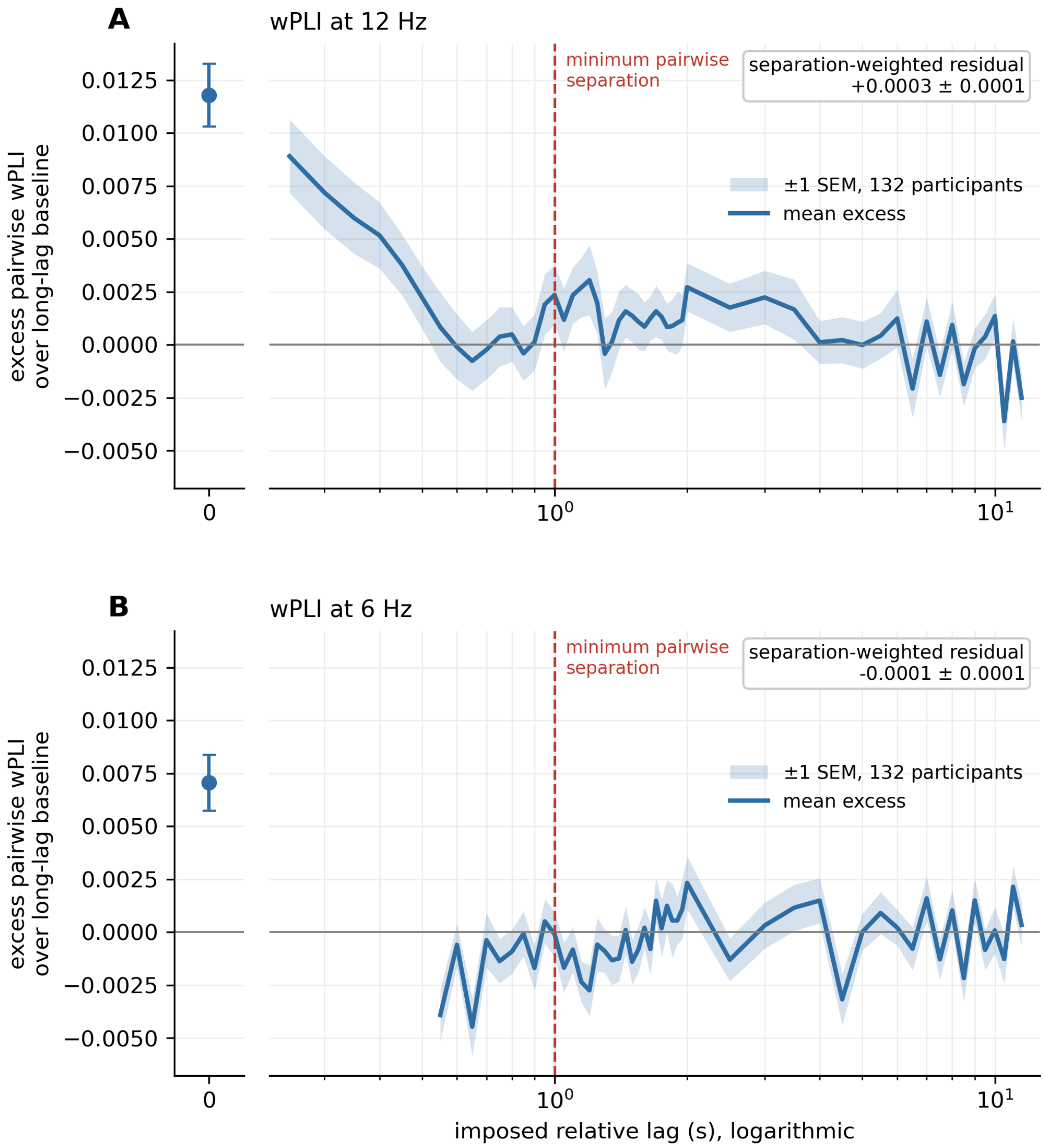

Figure S3: **Lag sensitivity of wPLI and assessment of the surrogate separation constraint.** Pairwise wPLI at **(A)** 12 Hz and **(B)** 6 Hz was recomputed after imposing a relative circular shift between channels. Each participant's curve was expressed as excess over their own long-lag baseline, defined as the mean across lags of 2–11.5 s. Lines show the mean excess across 132 participants, and shaded bands indicate ± 1 SEM. Separate points show the zero-lag excess with ± 1 SEM error bars; positive lags are displayed on a logarithmic axis. Curves begin at 0.25 s in (A) and 0.5 s in (B). Circular shifting changes relative phase as well as temporal alignment; these curves therefore assess empirical lag sensitivity rather than directly measuring the persistence of coupling. Excluding shorter lags does not eliminate phase-rotation effects at the evaluated lags. The dashed red lines mark the 1 s minimum pairwise circular separation used in the surrogate construction. Annotations report the excess averaged over the evaluated portion of the pairwise separation distribution produced by that construction: separations up to 11.5 s, approximately 90 % of the distribution, with weights renormalised. The resulting mean $\pm$ SEM across participants was $+0.0003 \pm 0.0001$ at 12 Hz and -0.0001 ± 0.0001 at 6 Hz. These small separation-weighted residuals support the selected constraint relative to the long-lag reference, but do not exclude dependence that persists at the reference lags. Separations between 11.5 and 13 s were not evaluated.
